# Operational Markov matrix formulation for structures in continuum plasma models

**DOI:** 10.1038/s41598-026-44059-6

**Published:** 2026-04-11

**Authors:** Nidhi Panday, Devendra Sharma

**Affiliations:** 1 Institute for Plasma Research, Bhat, Gandhinagar, Ahmedabad, 382428 Gujarat India; 2 Homi Bhabha National Institute, Training School Complex, Mumbai, 400094 India

**Keywords:** Markov-chain, Fluid-model, Plasma transport, Scrape-off layer, Structure formation, Monte-Carlo, Engineering, Mathematics and computing, Physics

## Abstract

Physical systems supporting stationary-structures allow translation of their mathematical formulations in to stochastic models. This paper presents the application of Markovian nonuniform generation formulation to achieve, for the first time, the analytically inaccessible flow configurations by letting the continuum fluid model to translate into a stochastic model of the Markovian class. The developed procedure achieves solutions in the infinitely large number limit of realizations, however completely free-from any computational realizations of the random variables. Fundamental properties of elements underlying this translation are developed by treating the representative, yet highly application relevant, case of plasma transport equilibria which solve the continuum fluid model of the target-bound plasma flow in the open magnetic field line region of fusion devices. Operational Subcomponent Markov Matrices (SMM), generating stationary structures of plasma flow, are constructed for the first time and quantitatively verified against the properties of SMMs systematically defined under the Markov-Chain Monte-Carlo (MCMC) formulation. The equivalent stochastic solutions simulated by a direct Markovian Monte-Carlo Simulation code MMSIM are validated against the corresponding structures produced by the developed SMMs in a procedure free-from numerical realizations of random variables. Benchmark of standard cases with the analytical solutions is presented followed by more general analytically inaccessible structures relevant to SOL configurations of fusion devices, demonstrating the capacity of SMM-MCMC based formulation to validate the general transport equilibria obtained from the associated stochastic simulations.

## Introduction

From simulating customer sales patterns for predicatively (and profitably) stabilizing the global supply chains^1,2^ to ensuring closely linked financial stability of the bourses^3,4^, or simply speeding up our internet searches^5,6^, the stochastic methods, best at *simulated annealing*^7^, remain center stage. Stochastic approach to deterministically recoverable equilibria of many physics models is possible by translating them, to some abstractness, into principles of Markovian processes underlying multivariate or combinatorial optimization^8^. In order to provide a brief introduction, consider, on the lines of Kirkpatrick et al.^8^, the iterative approach to selecting a minimum energy combination from a large pool by swapping, after each iteration, the old selection with new if it is discovered to have smaller energy functional. The availability of energy function or an equivalent optimization criterion is often the characteristics of the modeled physical systems and such problems fall in a special Markovian subdomain discussed further below. Such a search must overcome the possibility of getting trapped into local minima and a careful *annealing schedule* is called for. The question then is, can with an additional set of instructions, that physical problems might offer, the search be prevented from trapping in the local minima? and can it be rather free from large number of realizations of random variables? If formally in the Markovian domain, the answer to both question may be affirmative as a sophisticated translation of the models becomes possible into a universal formulation, as the mathematical elements of this transition can be generated and their Markovian character can be verified as done in the present paper with an application. Not uncommon, in the modern age of artificial intelligence (AI), that computational procedures are tasked with solving complex models with procedures free from realization of random variables to converge to optimum solutions. It is however rather unlikely that such translation of the continuum fluid model came into play any time you adopted the quickest available AI method among the prevailing ones to compute what flow structure emerges as the solution of a complicated fluid flow system, having arbitrary source and sink profiles. Since the fluid model has governing equations, a narrow pool of possibilities to search from does exist and the problem therefore is Markovian, however a translation of this model into the universal formulation, analogous to what your day-to-day queries to AI are piped into, requires constructing the Markovian matrices and need for realization of random variables is done away with.

Now, one might wonder why the continuum fluid model needs substitute for the Monte-Carlo procedure when it may not be the first choice for solving deterministic continuum structure formation problems of present interest. We however find that very sophisticated applications exist that do exclusively require stochastic or Monte-Carlo procedure for deterministic continuum model problems, especially when the complexities of the system go far beyond the reach of conventional solution procedures. Such examples come from challenges associated with handling the fourth state of the matter, or the plasma, that has its distributions modeled as continuum fluid in various regimes. The dynamics of this abstract fluid is studded with strong-nonlinearities and very complex geometries to solve for, especially in a magnetic confinement fusion reactor. Benefits can only be imagined if the universal translation underlying methods to solve Markovian class of optimization problems could be leveraged to solve the continuum model for generating structures in this system^9–13^. The first-ever development and application of the procedure to non-trivial cases of a 1-dimensional stable target-bound plasma flow is presented, which remains unchanged for higher dimensions and generalizes into multi-species and multi-dimensional cases merely by the multiple or nested calls to the fundamental procedures developed in the formulation developed here. The demonstration of dimension independence and application to higher dimensional domains remain subjects of advance steps in research, beyond our present scientific illustrations.

At this juncture it is perhaps appropriate to dive deeper into associated technicalities. In the standard context of stochastic generation of structures as systematically outlined by Sinclair and Sinclair et al.^14,15^, the uniform generation can be seen as a way of stochastically exploring a large set of combinatorial structures and constructing typical representatives of it. Non-uniform generation occurs in the mathematical modeling of physical systems where the structures are valid system configurations each of which has a weight which depends on its *energy*. Meaning, the principles are available to successively (iteration-wise) converge to structures of highest weight. It is this latter category of the systems which lands itself to the sophistication offered by the Markovian formulation over the conventional Monte-Carlo procedures. The question of existence of computationally efficient generational procedure for a given class of structures was addressed by Sinclair^15^. In this study we validate existence criteria outlined by Sinclair for continuum fluid model of a plasma and show the existence of an efficient procedure which is therefore guaranteed to generate solutions. We then present the first ever application of the procedure to generate the unique stationary solutions valid for magnetically confined plasmas. Although the conventional random variable based direct Monte-Carlo (MC) simulations remain leading tool to analyze complex plasma configurations^16–19^ because of their ability to handle shaped boundaries and faster convergence, in more specific terms their capacity is limited in exploiting standard matrix theory procedures far strongly optimized for using modern supercomputing paradigms^20^ and producing solutions in statistical limit, i.e., in the limit of random variable ensemble size approaching infinity. The stochastic Markovian simulations stand apart and lend themselves to matrix based solution procedures^21,22^, they however remain poorly adopted for solving the continuum models of structure formation, particularly in plasmas as treated here. One essential step in this procedure is to translate dynamical fields into the subcomponent transition matrices under the formal Markov-Chain Monte-Carlo (MCMC) formulation^14,15,21^.

In more quantitative terms, this study presents the first ever construction of working Markov matrices corresponding to the subcomponents of a quasineutral plasma transport model, having direct application, for example, in accessing stable solutions in the edge of a tokamak or its SOL region mapped to a 1-dimensional slab with planar boundaries like those described by Stangeby^23^ in order to implement the standard edge plasma fluid model of Braginskii^24^. The Markov matrices are constructed for dynamical subcomponents of the model associated with a field aligned isothermal plasma transport of the components, or individual species, of the plasma. Reduction of results, in the special case of the uniform source, is noted to duly produce the standard analytical solutions of the model^23,25,26^. The general solutions beyond the capacity of simple analytical treatment which are simulated by a direct Monte-Carlo code MMSIM are validated using the developed subcomponent matrix based MCMC procedure. The reconstruction, free-from random variable realizations, corresponds to statistical limit of MC, or the limit of infinitely large number of realizations.

In major illustrations of conformity with the criteria outlined by Sinclair^15^, it is established that the higher powers of Subcomponent Markov Matrices remain operationally equivalent to those converged from a series of recursive operations of the structures on the single-step subcomponent matrix^15^, and that all rows of higher power of the SMM attain uniformity, each identically representing the general solution. The agreement is shown between all the MCMC solutions and conventional MC results, including in the advance cases where analytical solutions remain inaccessible, specifically, the cases having nonuniform source/driver with and without symmetry with respect to boundaries of the one dimensional physical domain.

## MCMC paradigm for stochastic solution formalism

Continuum systems with smooth distribution of solutions allow stochastic or semi-stochastic random sampling of the solutions, to the accuracy determined by the number of realizations of random process. For clarity on the terminologies used in the rest of this paper, the generally accepted distinction between a conventional Monte-Carlo (MC) procedure and the Markov-Chain Monte-Carlo (MCMC) is followed^21^. Whereas the MCMC has a guaranteed stationary distribution of sequence of values generated during the random sampling of the integrand in an averaging process by virtue of an autocorrelated sampling process, the MC uses rather statistically independent sampling^15^. By this definition, though most stochastic approaches to deterministic or semi-deterministic problems remain MCMC, the strength of their MCMC character (over MC character) may be quantified by the extent of leveraging the correlations specific to MCMC rather systematically and free from numerical realizations of random-variables. Following this notion, a conventional stochastic approach relying on realization of random-variables^16^ rather than implementing the MCMC subcomponent matrix formulation developed here is addressed in rest of this paper simply as MC.

Addressing application to a multi-field physical transport model^23,24^, for example that of an isothermal field aligned plasma flow considered in this study, this sophistication is formulated and implemented over multiple dependent field variables, or subcomponents of MCMC. While their dynamics are interlinked by the model equations, it is allowable for individual subcomponents to have mutually independent rules, coefficients, and source distributions, hence distinct subcomponent matrices governing them.

Adopting the eigenvalue based formulation followed by Sinclair^15^, we let the (stationary) distribution of a subcomponent (its infinite ensemble elements $\chi$ sampling a domain having *N* nodes) denoted by a row vector $\pi ^{(t)'}$ such that $\pi _{i}^{(t)}=\textrm{Pr}(\chi _{t}=i)$. The initial distribution is $\pi ^{(0)'}$ and an initial state in an equivalent MC evolution is $\pi _{i}^{(0)}=1$ for some $i\in [N]$ which would evolve according to a transition matrix,

1 $$\begin{aligned} P=(p_{ij})_{i,j=1}^{N}, \end{aligned}$$

where $p_{ij}=\textrm{Pr}(\chi _{t+1}=j|\chi _{t}=i)$ is the probability of transition from state *i* to state *j* over a single step within the domain, independent of *t*. For the evolution to be formally MCMC the matrix *P* is non-negative and stochastic, i.e., its row sums are all unity. Among its main Properties,

1. A *t*-fold recursion $\pi ^{(t)}P=\pi ^{(t+1)}$ of the operation is such that $\pi ^{(t+1)}=\pi '$ for $t\rightarrow \infty$, i.e., multi-fold recursions result in the stationary structure, independent of initial value of *t*.
2. An *s*-step transition matrix $P^{(s)}$ can be obtained obeying,
  2 $$\begin{aligned} P^{(s)}=P^{s}=\left( p_{ij}^{(s)}\right) _{i,j=1}^{N}, \end{aligned}$$
  where $P^{s}=\Pi _{i=1}^{s}P^{i}$ and $p_{ij}^{(s)}=\textrm{Pr}(\chi _{t+s}=j|\chi _{t}=i)$, independent of *t*.
3. For $P^{(s)}$ to be associated with a stationary distribution $\pi '$ of the subcomponent $\chi$ in domain [*N*],
  3 $$\begin{aligned} {\lim _{s\rightarrow \infty }}p_{ij}^{(s)}=\pi _{j}. \end{aligned}$$
4. If (3) is satisfied it is ensured that,
  4 $$\begin{aligned} \pi ^{(t)'}=\pi ^{(0)'}P^{t}\rightarrow \pi ^{t} ~~\textrm{as}~~ t\rightarrow \infty , \end{aligned}$$
  meaning that the left eigenvectors of $P^{t}$ having eigenvalues unity are the stationary structures in $t\rightarrow \infty$ limit.

Properties 1 - 3 are now shown to be possessed by a set of subcomponent matrices constructed for a physical model describing the isothermal plasma flow developed along a field-aligned (1-dimensional) SOL. Before presenting results and analysis we present the SOL model for which the operational SMMs will be constructed. In the first part of analysis, following their construction, the capacity of matrices is shown to regenerate a subcomponent structure when its complement subcomponent is adopted from the known analytical solutions. In the second part, cases are treated for which no correct/analytical solutions are either provided or available of the physical model, forcing the choice of initial SMMs to be arbitrary and mutually inconsistent. The global convergence to the solutions by a MCMC based MMSIM code simulations however makes mutually consistent SMMs available for the validation by final SMMs to progress. The MCMC procedure outlined above thus validates SMMs against the Properties 1 - 3 as well as the numerically *converged* MC simulation results.

### The SOL plasma flow model

The idea of a model being available formally makes present procedure to belong to the class of non-uniform generation. As described by Sinclair^15^, this remains analogous to well known stochastic technique of the combinatorial optimization, namely, the simulated annealing which involves implementing a careful *annealing schedule* in order to escape local optimization minima. In our presentation, however, the MCMC procedure guides the solutions directly to the unique physical solutions of the model, across the iterations, provided - the stability criterion for the physical plasma flows in the model. We begin by considering the basic fluid-dynamical model of a plasma isothermally flowing into a planar absorbing boundary. Specifically, the Scrape-off-Layer (SOL) of a tokamak where a quasineutral plasma of local density *n* and electron temperature $T_{e}$ steadily flows with local flow velocity *u* along the confining magnetic field into a planar target, namely, a limiter or a divertor^9,33^,

5 $$\begin{aligned} \partial _{t} n+\partial _{x} (\Gamma _{p})= & S_{p},\end{aligned}$$

6 $$\begin{aligned} \partial _{t} (mnu)+\partial _{x} (m\Gamma _{m})= & -\partial _{x} (nkT), \end{aligned}$$

where subscript *t* and *x* denote derivatives with respect to time and parallel length *x* respectively, $S_{p}$ is the net plasma source, *k* is the Boltzmann constant, $T=T_{e}$ is the uniform electron temperature with ion fluid assumed to be cold ($T_{i}\rightarrow 0$). The energy or heat transport equation is excluded in favor of the model (5)-(6) still reliably representing the isothermal or sheath limited operational regime of the SOL plasmas (see Rognlien et al. illustration in Fig. 3 of Ref. ^33^) and allowing, in turn, to limit to only two subcomponents of the model. The mass and momentum fluxes $\Gamma _{p}$ and $\Gamma _{m}$ are combinations of both deterministic and stochastic contributions to them,

7 $$\begin{aligned} \Gamma _{p}= & nu-D_{p}\partial _{x} n,\end{aligned}$$

8 $$\begin{aligned} \Gamma _{m}= & nuu-D_{m}\partial _{x} (nu). \end{aligned}$$

Note that the longitudinal (electrostatic) ambipolar electric field ($E=-\partial _{x}\phi$) of the plasma is eliminated when *E* satisfies $\partial _{x} E=4 \pi e(n_{i}-n_{e})$ and electron density $n_{e}$ is well approximated by the Boltzmann relation $n_{e}=n_{0}\exp {(-e\phi /kT_{e})}$ where $\phi$ is electrostatic potential and *e* is magnitude of electronic charge. This system is a suitable example of a single physical component (single quasineutral fluid) system with multiple dynamical subcomponents, namely, density and momentum, requiring to be treated. First non-trivial case of equilibrium in this regime is of a nondiffusive ($D\rightarrow 0$) stable plasma flow in to an absorbing boundary, requiring, upon imposing the stability criterion, the plasma outflow velocity value to marginally exceed the ion sound speed $c_{s}=\sqrt{kT_{e}/m_{i}}$ at the boundaries of the domain^11^. For quasineutral plasma flow the system length $x_{s}$ remains the natural length scale. Since the system is resolved by moderately large number of points, the resolution, or the separation between two consecutive points $L=x_{s}/N$ is treated as normalizations for length in the present analysis. For upstream plasma density $n_{0}$ comparable to a tokamak device and $x_{s}$ comparable to connection length therein, the electrostatic plasma sheath forming at the boundaries of the system is vanishingly thin as compared to *L* and scales with the plasma Debye length, $\lambda _\textrm{D}=\sqrt{kT_{e}/4\pi n e^{2}}\ll L$. Hence *L* and $c_{s}$ remain the natural length and velocity scales of the variations in the profiles and the same are used as normalizations in the present analysis.

## Results

### MCMC subcomponents matrix construction for stable SOL plasma flow model

**Figure 1:**
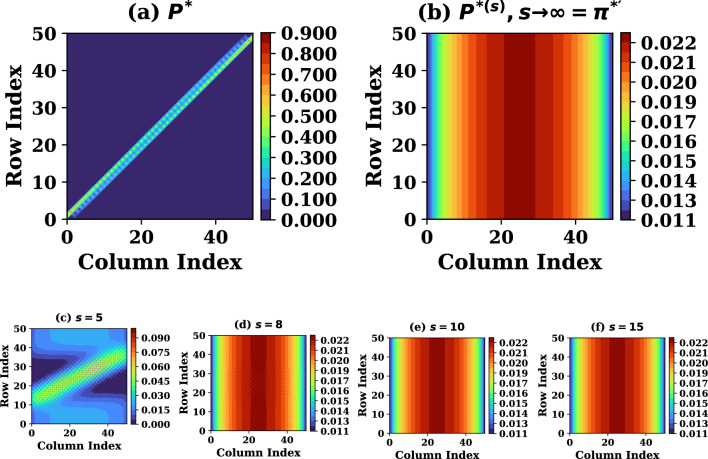
The density SMM (**a**) for single step $P^{*}$ and (**b**) for multi-step $\pi ^{*'}$, of size $N\times N=50\times 50$ from analytical solutions of the model (5)–(6). Frames (**c**)–(**f**) present $P^{*(s)}$ at intermediate *s*.

We now set out to construct the subcomponent Markov matrices having real eigenvalues and completely determinable eigenvectors. The full set of subcomponent matrices $$P=(P^{*},P^{**})$$ is determined following (1) which defines its individual elements. More specifically, the transition probability value is proportional to normalized convective flow velocity out of the $$i^{th}$$ cell. The rest of the velocity fraction, from total probability, is distributed isotropically so as to cause no net flow in absence of macroscopic gradients, ruling out the risk of particle accumulation when flow velocity vanishes. This is in accordance to irreducibility of the Markov chain of concern. Further, we limit the difference $$|i-j|\le 1$$ and use the distribution of the velocity variable *u*, a version of which is known at each stage of its refinement, to assign *N* values of $$p_{ij}$$ for each row index *i*, treating this *u* as stationary, *a priory*. In order to prevent the exterior as terminating state for the chain, the values of first and last row of *P* are determined by the profile of the source, which balances the sink for the stationary states. This procedure yields the initial form of *P* as a reasonably sparse $$N\times N$$ matrices. The first member of the SMM set, $$P^{*}$$, is plotted in Fig. 1a using $$N=50$$ $$(=x_{s}/L)$$ as obtained for a special case where source is uniform and analytical solutions of the model (5)-(6) are known as (9) and (10). This relaxes the need for an iterative refinement of set $$\pi =(\pi ^{*},\pi ^{**},\pi ^{***},\dots )$$ to determine physically consistent set *P* for this case, as done further below for advanced cases. Note that the initial imposition of $$|i-j|\le 1$$ does not restrict the procedure to shortest jumps, as the subsequently computed $$P^{*{(s)}}$$ facilitates jumps of arbitrarily length $$\le s$$. Additionally, the solutions represent stationary states if the full set of subcomponent matrices $$P\equiv (P^{*},P^{**}\dots )$$ does not further evolve over subsequent iterations.

### Validation of basic SMM properties

In this first part, the analytical solutions of the model (5)-(6) known in the uniform plasma source limit are used to validate the constructed SMMs $$P=(P^{*},P^{**})$$. The same *P* is subsequently used also to validate the Properties 1 - 3 such that it is proven to be authentic set of SMMs for a physical system, corresponding to a tokamak SOL transport equilibria in statistical limit of the conventional MC simulation procedure.

#### Determination of eigenvectors corresponding to SOL plasma profiles

**Figure 2:**
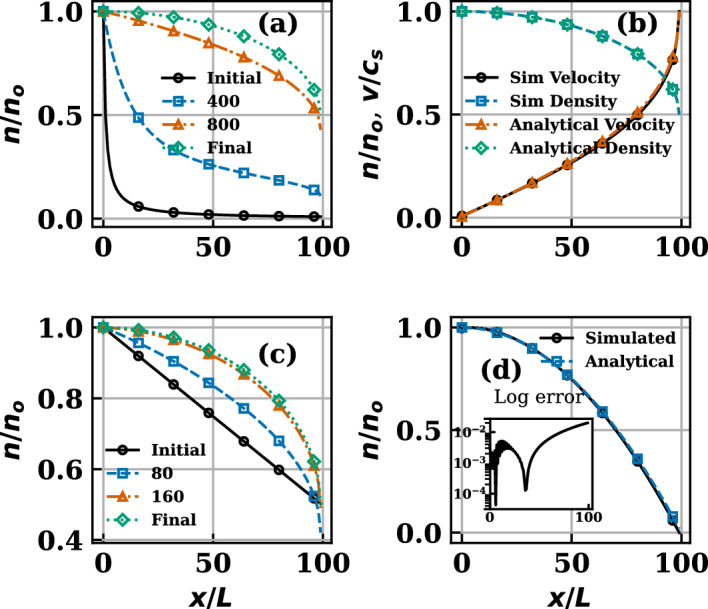
orange(**a**) and (**c**) The recursive (with increasing *s*) development of the initial density $\pi ^{*(0)}$ and convergence in to final stationary solution $\pi ^{*'}$ of (5) - (6), from two distinct initial profiles (black lines in (**a**) and (**c**), respectively), consistent with Property - 1. (**b**) Analytical solutions of (5)–(6) for $D\rightarrow 0$. (**d**) Comparison between $\pi ^{*'}$ and analytical solutions of (5)–(6) in pure diffusive transport limit.

The continuum analytical solutions of the model (5)-(6), as derived in Appendix B, corresponding to a uniform source generated plasma density *n* and flow velocity *u* in the spatial domain $[-x_{s},x_{s}]$ are,

9 $$\begin{aligned} u= & c_{s}\left[ \frac{x_{s}}{x}-\sqrt{\left( \frac{x_{s}^{2}}{{x}^{2}}\right) -1}\right] ,\end{aligned}$$

10 $$\begin{aligned} n= & n_{0}\left( \frac{c_{s}^{2}}{c_{s}^{2}+u^{2}}\right) , \end{aligned}$$

respectively. As discussed above, when the exact solutions are known, the multi-step matrices $$P^{(s)}_{s\rightarrow \infty }$$ are readily constructed and the solutions employed in their construction are verified to be specific left-eigenvectors of these subcomponent matrices. The numerically computed left-eigenvectors $$\pi _{i}\equiv (\pi _{i}^{*},\pi _{i}^{**})$$ of the independently constructed single-step subcomponent matrices $$P=(p_{ij})_{i,j=1}^{N}$$ from the solutions (9) and (10) are graphically shown in Fig. 2b to reproduce the density and velocity profiles, respectively, to good accuracy by fixing $$|x_{s}|=100 L$$.

In order to validate Properties 1 - 3 of the SMMs constructed in this trivial case with known analytical solutions, the initial distribution $\pi ^{*(0)}$ was varied from its known analytical profile (10) and recursion prescribed by Property-1 are made recovering the solutions agreeing with the analytical solutions after the number of recursions depending on the deviation of $\pi ^{*(0)}$ from $\pi ^{*'}$. Two choices for initial varied $\pi ^{*(0)}$ profile are additionally plotted in Fig. 2a,c, with its improving versions at intermediate recursions before it converges to the known analytical solutions $\pi ^{*'}$. The subsequent Properties 2 - 4 are validated by the surface plots of SMM presented in Fig. 1 explained as follows: (1) for increasing powers of $P^{*}$ it becomes stationary after $s \sim 8$ as can be seen in Fig. 1d which is a direct proof of property 2. (2) At sufficiently high powers, all rows of $P^{*}$ converge to the same distribution $\pi ^{*'}$, as illustrated in Fig. 1b, property 3 is therefore satisfied because every row of the stationary matrix identically represents $\pi ^{*'}$. (3) Iterative operation of the stochastic transition matrix on an arbitrary initial state drives the system toward the stationary distribution, as demonstrated in Fig. 1b, which is a direct consequence of property 4.

Note, however, that this case, despite independently allowing to validate Properties 1 - 4 of the matrices $$\pi ^{*'}$$ and $$\pi ^{**'}$$ with the help of either one or both of them being analytically known for constructing *P*, is rather trivial. In the general case of a physics problem being solved with unknown solutions, the $$\pi ^{*(0)}$$ and $$\pi ^{**(0)}$$ although produce $$\pi ^{*'}$$ and $$\pi ^{**'}$$ by higher recursions of initial *P*, this pair essentially remains inconsistent unless a physical convergence is achieved (either by SMM-MCMC iterations or by a corresponding MC simulation code, as in the present case) making available a physically consistent and globally converged choice with respect to the plasma model (5) - (6). This rather non-trivial exercise is done and presented further below treating the general cases of transport equilibria.

Before closing this section we present solutions from an alternate set of *P* which is constructed in the limit of pure diffusive plasma transport, having no deterministic contribution *nu* in $\Gamma _{p}$ given by (7), such that $\pi \equiv (\pi ^{*}_{i})$. The numerically computed stationary eigenvector ($\pi ^{*'}_{i}$) is compared in Fig. 2d for this special case with the corresponding analytical density profile which is the solution of pure diffusion equation,

11 $$\begin{aligned} n= & n_{0}\left( 1-{x^{2}}/{x_{s}^{2}}\right) . \end{aligned}$$

in the domain $[-x_{s},x_{s}]$ with $|x_{s}|=100~L$,

### Computation of general transport equilibria

Having validated the general Properties 1 - 3 using the SMM developed for the standard case with known analytical solutions, we now present construction and application of the SMM to the general cases of transport equilibrium with no known solutions available. In the present study they are obtained both by iteratively converged conventional MC simulations done using the code MMSIM and by the equivalent SMM-MCMC procedure. The analysis, in turn, also serves as a benchmark of the conventional special Markov-specific direct MC simulations procedure underlying the MMSIM code. The SMM-MCMC procedure implemented by us, to these advance cases, treats the transition of positive and negative momentum ensembles of chains (fluid parcel trails) separately, so as to construct net density and momentum in the states by their weighted superposition. This is done by assigning appropriate values of first and last rows of *P* such that they reconnect the points of chains’ exit from the physical domain to its interior, in numbers proportional to respective local particle and momentum sources. This rather non-unique scheme adopted by us limits the required number of SMMs to its original value of 2, preventing excess or non-essential computations in comparison to the case if one density and two momentum SMMs were considered as a result of acknowledging separation between positive and negative momentum ensembles. Note that, as originally mentioned, this choice remains non-unique and is one among several equivalent options possible for partitioning scaled transition probabilities that are in compliance with the general SMM formulation. Following this special choice of probability partition, the information of the source profiles from the model only enters the first and last row of respective *P*, essentially preserving the mutually identical structure of element values in the interior of all the SMMs. It is important to mention that while certain of these non-unique partition options may not be available based on the physical model and its dimensionality, certain others may become additionally available. A flow-chart of the numerical procedure followed is presented in Appendix B.

**Figure 3:**
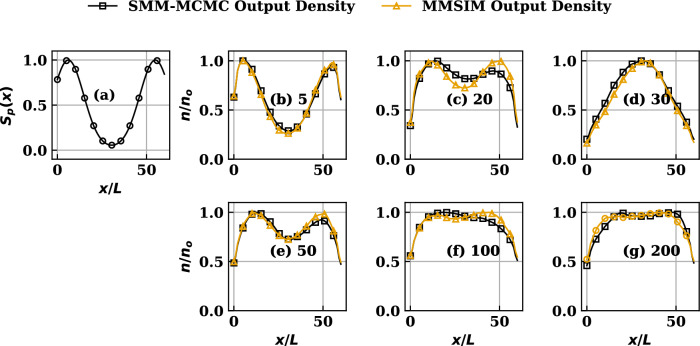
(**a**) The nonuniform symmetric source profile. (**b**–**g**) Comparison of MMSIM simulated density profiles reproduced by the SMM-MCMC procedure at various iteration numbers (written besides the label on each subplot from (**b**–**g**)) for source profile (**a**).

**Figure 4:**
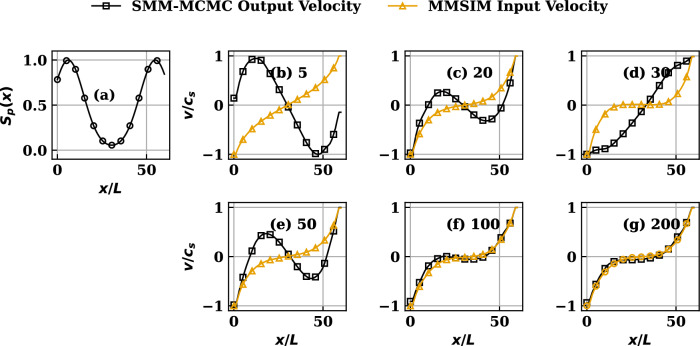
(**a**) The nonuniform symmetric source profile. (**b**–**g**) Comparison of MMSIM simulated velocity profiles reproduced by the SMM-MCMC procedure at various iteration numbers (written besides the label on each subplot from (**b**–**g**)) for source profile (**a**). Note that the MMSIM profiles correspond to the input provided to the code during the indicated iteration while the SMM-MCMC profiles correspond to output from the code from that iteration.

The two sets of general case MC simulations, performed, reproduced with SMM-MCMC procedure and analyzed here, include: solutions with a nonuniform source distribution having (i) symmetry and (ii) asymmetry with respect to the boundaries/center of the simulation domain. This is achieved by using,

12 $$\begin{aligned} S=S_{0}+\sum _{i=1}^{k}A~\exp {[-(x-x_{i})^{2}/\alpha x_{s}^{2}]}, \end{aligned}$$

which allows source profiles to be peaked at limited set of locations $(x_{1},\dots , x_{k})$, distributed either symmetrically or asymmetrically about the center of the domain, over a uniform offset value $S_{0}$. Considering that $\int _{0}^{x_{s}} S dx$ balances the net outflow from the boundaries, the ratio $A/S_{0}$ and $\alpha$ are only free parameter for the source distribution, which are set to be 200 and 0.04, respectively, for all cases.

#### Symmetric non-uniform source distribution

The symmetric source profile, achieved by setting $$k=2$$, $$(x_{1},x_{2})=(0.1x_{s},0.9x_{s})$$ in (12) and used for this case, is plotted in Figs. 3, 4a and results showing comparison between MMSIM generated (yellow) and SMM-MCMC generated (black) density profiles are plotted for increasing number of MMSIM iterations in Fig. 3b–g, respectively. The corresponding velocity profiles are plotted in Fig. 4b–g with the same color scheme. In each case, the analytical solutions of uniform source case presented above are used as the initial structures ($$\pi ^{*'},\pi ^{**'}$$) in the simulations for $$N=60$$. Note therefore that for profiles in Fig. 3b, the $$\pi ^{*}$$ from simulations and SMMs agree, however $$\pi ^{**'}$$ show a strong disagreement as a result of inconsistency with respect to the physics model which has nonuniform source (Fig. 4a) in its version solved by the MMSIM code simulation. However an iteration-wise improvement in the output of the MMSIM code is visible as the both $$\pi ^{*'}$$ (density) and $$\pi ^{**'}$$ (velocity) begin to converge as a pair and no longer undergo further modification after sufficient number of iterations shown from Figs. 3b–g and 4b–g, respectively. In case of velocity plots (Fig. 4), the MMSIM profiles correspond to the input provided to the code during that iteration while the SMM-MCMC profiles correspond to output from the code from that iteration. In case of density plots (Fig. 3), however, both are output density profiles from the respective codes. With the help of such choice for plotting, it is effectively highlighted that how much input and output have mutually differed during the initial iterations despite the initial guess provided has been quite close to what is expected of convergence. A slow convergence over large number of iterations is result of a rather careful (less relaxed) iterative update of the profiles ensuring stability.

**Figure 5:**
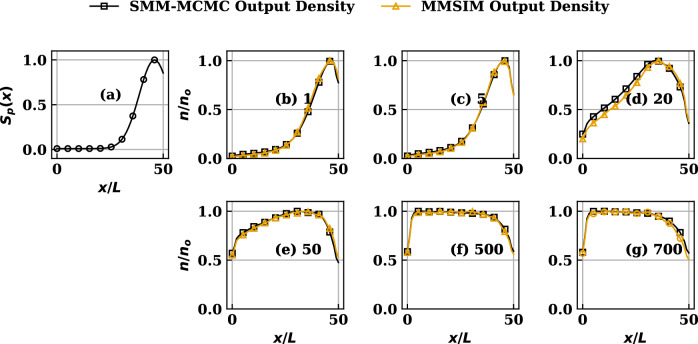
(**a**) The nonuniform asymmetric source profile. (**b**–**g**) Comparison of MMSIM simulated density profiles reproduced by the SMM procedure at various iteration numbers (written besides the label on each subplot from (**b**–**g**)) for the source profile (**a**).

**Figure 6:**
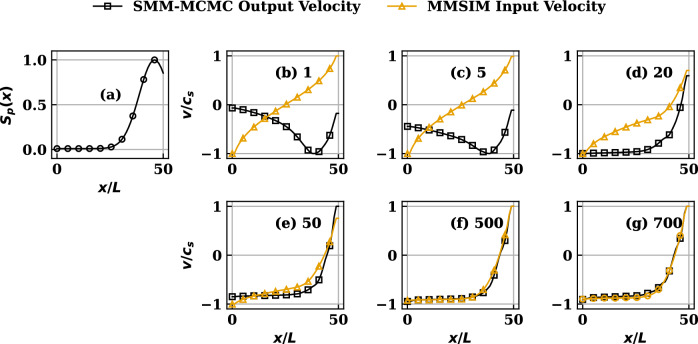
(**a**) The nonuniform asymmetric source profile. (**b**–**g**) Comparison of MMSIM simulated velocity profiles reproduced by the SMM procedure at various iteration numbers (written besides the label on each subplot from (**b**–**g**)) for the source profile (**a**). Note that the MMSIM profiles correspond to the input provided to the code during the indicated iteration while the SMM-MCMC profiles correspond to output from the code from that iteration.

**Figure 7:**
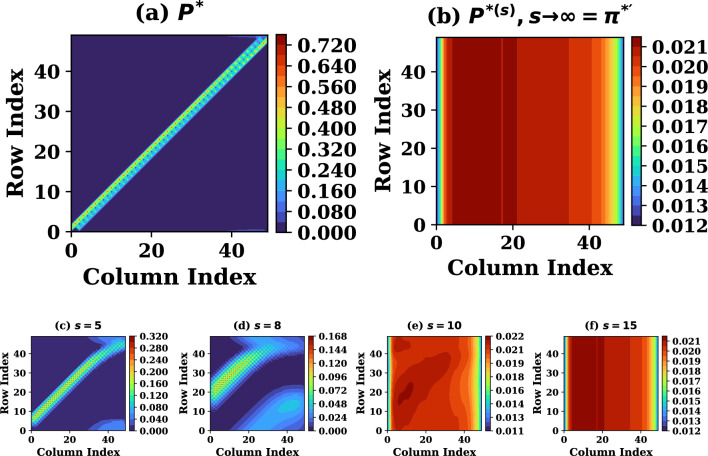
The density SMM (**a**) for single step $P^{*}$ and (**b**) for multi-step $\pi ^{*'}$, of size $N\times N=50\times 50$ from solutions of the model (5)–(6) using asymmetric source profile plotted in Fig. 5a. Frames (**c**–**f**) present $P^{*(s)}$ at intermediate *s*.

The reproduction of the MC simulation solutions from the SMM generated eigenvectors validates the direct MC simulation procedure and can, in turn, provide quantitative validation of the general MC simulation results for mutual consistency among output subcomponents ($$\pi ^{*'},\pi ^{**'},\pi ^{***'},\dots )$$ in the regimes where no alternate solution procedure is likely to be available.

In accordance with Properties 1 - 4 of the SMMs, both recursion-wise and power-wise convergence to stationary structures is confirmed to be achieved and the matrix $P^{*}$ for this case remains qualitatively similar to that of the analytical solution case which was presented by plotting it the Fig. 1a,b. While all the rows of the matrix $P^{*}$ approach the symmetric profile of $\pi ^{*'}$ at higher power of $P^{*}$ corresponding to symmetric source used in this case, the intermediate powers of $P^{*}$ are plotted in small frames in the bottom row of the Fig. 1 showing gradual transformation of powers of $P^{*}$ in to $\pi ^{*'}$.

#### Asymmetric non-uniform source distribution

Considering realistic SOL plasma flow scenarios and often present localized ionization sources that lead to up-down and inboard-outboard asymmetries in them, we present a case with non-uniform source profile. The cases with asymmetry also address the potential application of the procedure to simulate plasma transport in complex situations, for example in a tokamak SOL with diverted plasmas where the transport in inboard and outboard divertor regions may be strongly dissimilar^27^. To this end, we examine a case of an asymmetric plasma source which is localized in the *recycling* region of SOL, or away from the core region of the equilibrium and uneven on both the divertor legs. The source profile with localization close to one of the boundaries is achieved by setting $k=1, (x_{1})=(0.9x_{s})$ in (12) and plotted in Figs. 5, 6a which produces asymmetric solutions. The corresponding comparison between MMSIM generated (yellow) and SMM-MCMC generated (black) stationary output density profiles is done by plotting them for increasing number of MMSIM code iterations in Fig. 5b–g, respectively, for $N=50$, the parameter *N* represents the number of accessible states of the system, changing *N* alters the size of the stochastic transition matrix, which has dimension $N \times N$, and thereby directly impacts the computational cost, as the complexity of the associated algebraic operations scales with the matrix dimension. Also, since the total probability is shared among the accessible states, increasing *N* leads to a progressively more uniform probability distribution. As the discreteness of the distribution decreases, the resulting solutions correspondingly approach the continuum limit and become more accurate. The corresponding velocity profiles are plotted in Fig. 6b–g with the same color scheme. Like the symmetric source cases, the initial seed profiles used are the analytical solutions of uniform source (9) and (10). An iteration-wise improvement in the output of the MMSIM code results is once again visible as the profiles of density and flow velocity begin to converge and no longer undergo further modification following a finite number of initial iterations. Once gain, in case of velocity plots (Fig. 6), the MMSIM profiles correspond to the input provided to the code during that iteration while the SMM-MCMC profiles correspond to output from the code from that iteration. In case of density plots (Fig. 5), however, both are output density profiles from the respective codes.

In accordance with Properties 1 - 4 of the SMMs, both recursion-wise and power-wise convergence to stationary structures is again confirmed to be achieved as presented by plotting matrix $P^{*}$ in the Fig. 7a,b. $P^{*}$ is stochastic in nature i.e. all its rows sum to unity, all the powers of a stochastic matrix are also stochastic in nature as they represent the transition probabilities for $n^{th}$ jump. All the rows of matrix $P^{*}$ approach a common asymmetric profile of $\pi ^{*'}$ at higher power of $P^{*}$ corresponding to asymmetric source (Fig. 5a) used in this case. The intermediate powers of $P^{*}$ are plotted in small frames in the bottom row of the Fig. 7 showing gradual transformation of powers of $P^{*}$ in to an asymmetric stationary profile $\pi ^{*'}$. We finally note that both MMSIM and SMM-MCMC might converge over similar number of iterations, however advantage of SMM-MCMC lies in the fact that the execution time, per iteration, for MMSIM remains way longer than that for SMM-MCMC, hence the overall computation time taken by the SMM-MCMC to approach convergence remains considerably smaller than the equivalent MMSIM simulations.

To summarize, the analysis and cases present above establish the applicability of the Markov formulation to *stationary* structures of the continuum fluid dynamics, which is in distinction from the routine applicability of Markov formulation to turbulent or particle dynamical stochastic processes^13,18,28–32^. The operational example of source-driven target-bound plasma flow, representing the SOL region of fusion devices, treated to establish the fundamental properties of the SMMs allow ready applicability of the results to benefit the present direct MC simulation efforts in SOL plasma modeling. The illustrations and validations open potential fresh avenues of exploring rather abstract structures supported by continuum model in plasmas and general physics.

## Discussion

Applicability of stochastic Markov matrix formulation based approach to an operational system is demonstrated by treating the stationary structures that are transport equilibria of a field-aligned stable quasineutral plasma flow to the planar targets. The plasma fluid model solved by these equilibria allows its translation in to a stochastic dynamical formulation under which the subcomponent Markov Matrices can be constructed. They relate to multiple dependent field variables associated with each plasma constituent or component which provide stationary solutions following systematic definition of the SMMs and determination its eigenvectors.

The outcome presented includes:

- construction of Markov matrices corresponding to dynamical subcomponents associated with a field aligned transport of the plasma components, or species. For the choice of quasineutral plasma as a single fluid, a set of two subcomponents corresponding to the density and momentum of the plasma fluid has been treated. Reduction of results, in the special case of the uniform source, is noted to duly produce the standard analytical solutions of the model^23,24^.
- The general solutions beyond the capacity of analytical treatment are simulated and then subjected to validation by the developed SMM based MCMC procedure. The validation used solutions as reconstructed using the subcomponent matrices and compared them with the solutions as originally simulated using an equivalent MC simulation code MMSIM that works by employing random variables for producing converged solutions to finite level of statistical fluctuations.
- The agreement is shown between all the MCMC solutions and conventional MC results, including in the advance cases where analytical solutions remain inaccessible, i.e., the cases with nonuniform source/driver either possessing symmetry or no symmetry with respect to the boundaries of the one dimensional physical domain.
- The SMM based MCMC procedure, free-from numerical realization of random variables, and associated noise is applied first to validate the fundamental properties of general subcomponent matrices, namely, (i) recursive operations on a single-step subcomponent matrix by an arbitrary initial eigenvector produces the stationary structure in large number of recursion limit. (ii) A multi-step SMM is operationally equivalent to the corresponding power of the single-step SMM. (iii) The multi-step SMM in infinite steps limit identically features, in all its rows, set of a unique eigenvector with eigenvalues unity, representing, in turn, the stationary structure. (iv) The multi-step SMM obtained in the infinite steps limit transforms an initial left operating eigenvector into the stationary structure.
- The structures are reconstructed by using the developed SMM procedure for both non-converged and converged sets of output of the equivalent Monte-Carlo simulation code MMSIM. The reconstruction of full set of output is however achieved only for the *converged* set of simulation output. The SMM procedure is thus shown to provide independent validation of the output of the conventional Monte-Carlo simulations.

Among the important future works is to advance the SMM-MCMC based procedure for application to larger numbers of plasma components as well as the associated dynamical subcomponents. In this context, it is worth mentioning that while the structure of the fundamental Markovian elements of the simulation remains unchanged for any application to higher dimensional domains. Even for two, three or higher-dimensional physical domains, the stochastic transition matrix which constitutes the kernel of the present method remains two-dimensional, i.e., of size $N \times N$ , where *N* is the number of states in the ensemble representation of the system. Increasing the physical dimensionality only increases *N*, thereby scaling the computational cost accordingly, as discussed above, the physical models involve additional novel measures while translating in to identical set of elements as essentially available in the 1-dimensional model. The demonstration of dimension independence and presentation of these novel aspects specific to higher dimensional physical domains (or hyper-spaces, e.g., phase-space) therefore remain subjects of advance steps in research, beyond our present scientific illustrations. Similarly the treatment generalizes into multi-species and multi-dimensional cases merely by the multiple or nested calls to the fundamental procedures developed in the formulation developed here, although the required multiplicity of iterations must be accounted for during such generalizations. Most enhancements thus relating to a higher capacity of the physics model that incorporates, for example, the more complex aspects of the plasma-neutral physics and self-consistently determines the plasma source in the SOL region, may also to be potentially addressed under the Markovian domain.

## Data Availability

Data that support the findings of this study have been submitted for journal’s reference. Also, The data that support the plots and findings of this paper are available from the corresponding author on reasonable request. However, due to other novel findings, authors won’t be able to make the raw data public.

## Appendix A

Here we derive the full expressions given in Eqs. (9) and (10) by solving Eqs. (5) and (6) for steady state, for a purely convective flow corresponding to a uniform source $S_{p}$ of plasma which supports plasma density *n* and flow velocity *u* profiles that obey,

13 $$\begin{aligned} \partial _{t} n+\partial _{x} (nu)= & S_{p},\end{aligned}$$

14 $$\begin{aligned} \partial _{t} (mnu)+\partial _{x} (mnuu)= & -\partial _{x} (nkT), \end{aligned}$$

and the conditions for steady state :

15 $$\begin{aligned} \frac{\partial n}{\partial t}= & 0,\end{aligned}$$

16 $$\begin{aligned} \partial _{x} (nu)= & S_{p},\end{aligned}$$

17 $$\begin{aligned} \partial _{x} (mnuu)= & -\partial _{x} (nkT), \end{aligned}$$

Equations (16) and (17) are combined to eliminate $\frac{\partial n}{\partial x}$ and after substituting $\frac{u}{c_{s}}=M$, where $c_{s}=\sqrt{kT_{e}/m_{i}}$, we obtain the following expression:

18 $$\begin{aligned} \frac{dM}{dx}= & \frac{S_{p}}{nc_{s}}\frac{(1+M^{2})}{(1-M^{2})},\end{aligned}$$

19 $$\begin{aligned} where, M= & \frac{u}{c_{s}}, \end{aligned}$$

On integrating Eq. (16) we get:

20 $$\begin{aligned} nu= & S_{p}x,\end{aligned}$$

21 $$\begin{aligned} n= & \frac{S_{p}x}{u}, \end{aligned}$$

Substituting n from Eqs. (21) to (18) and integrating, we obtain the following results:

22 $$\begin{aligned} u= & c_{s}\left[ \frac{x_{s}}{x}-\sqrt{\left( \frac{x_{s}^{2}}{{x}^{2}}\right) -1}\right] , \end{aligned}$$

23 $$\begin{aligned} n= & n_{0}\left( \frac{c_{s}^{2}}{c_{s}^{2}+u^{2}}\right) , \end{aligned}$$

## Appendix B

The pseudo-code (flow-chart) of the SMM-MCMC procedure is shown as follows:
